# Hyperkalemia in CKD: an overview of available therapeutic strategies

**DOI:** 10.3389/fmed.2023.1178140

**Published:** 2023-07-31

**Authors:** Davide Costa, Gemma Patella, Michele Provenzano, Nicola Ielapi, Teresa Faga, Mariateresa Zicarelli, Franco Arturi, Giuseppe Coppolino, Davide Bolignano, Giovambattista De Sarro, Umberto Marcello Bracale, Luca De Nicola, Paolo Chiodini, Raffaele Serra, Michele Andreucci

**Affiliations:** ^1^Department of Law, Economics and Sociology, University Magna Graecia of Catanzaro, Catanzaro, Italy; ^2^Renal Unit, Department of Health Sciences, “Magna Graecia” University of Catanzaro, Catanzaro, Italy; ^3^Department of Medical and Surgical Sciences, University of Bologna, Bologna, Italy; ^4^Department of Public Health and Infectious Disease, Sapienza University of Rome, Rome, Italy; ^5^Unit of Internal Medicine, Department of Medical and Surgical Sciences, University “Magna Graecia” of Catanzaro, Catanzaro, Italy; ^6^Renal Unit, Department of Medical and Surgical Sciences, University “Magna Graecia” of Catanzaro, Catanzaro, Italy; ^7^Department of Health Sciences, “Magna Graecia” University of Catanzaro, Catanzaro, Italy; ^8^Department of Public Health, “Federico II” University of Naples, Naples, Italy; ^9^Renal Unit, University of Campania “LuigiVanvitelli”, Naples, Italy; ^10^Department of Mental and Physical Health and Preventive Medicine, University of Campania “Luigi Vanvitelli”, Naples, Italy; ^11^Unit of Vascular Surgery, Department of Medical and Surgical Sciences, University “Magna Graecia” of Catanzaro, Catanzaro, Italy

**Keywords:** serum potassium, chronic kidney disease, RAAS-blockade, potassium binder, hypokalemic agents

## Abstract

Hyperkalemia (HK) is a life-threatening condition that often occurs in patients with chronic kidney disease (CKD). High serum potassium (sKsK) is responsible for a higher risk of end-stage renal disease, arrhythmias and mortality. This risk increases in patients that discontinue cardio-nephroprotective renin–angiotensin–aldosterone system inhibitor (RAASi) therapy after developing HK. Hence, the management of HK deserves the attention of the clinician in order to optimize the therapeutic strategies of chronic treatment of HK in the CKD patient. The adoption in clinical practice of the new hypokalaemic agents patiromer and sodium zirconium cyclosilicate (SZC) for the prevention and chronic treatment of HK could allow patients, suffering from heart failure and chronic renal failure, to continue to benefit from RAASi therapy. We have updated a narrative review of the clear variables, correct definition, epidemiology, pathogenesis, etiology and classifications for HK among non-dialysis CKD (ND CKD) patients. Furthermore, by describing the prognostic impact on mortality and on the progression of renal damage, we want to outline the strategies currently available for the control of potassium (K+) plasma levels.

## 1. Introduction

The increase in serum potassium (sK) levels is a hydro-electrolytic alteration that frequently occurs in patients with chronic kidney disease (CKD) (1, 2). This complication is associated with an unfavourable and life threatening prognosis due to its cardiotoxicity and increased mortality risk (3). The risk of hyperkalemia (HK) increases with CKD, and can compromise CKD patient safety (4). In addition, the frequency of HK increases with the use of drugs prescribed for their beneficial cardio-renal properties, namelyrenin-angiotensin-aldosterone system inhibitor drugs (RAASi), often counteracting their clinical benefit in CKD patients (5). In hospitalized patients, CKD and prolonged HK are independent negative prognostic factors. The increase in K+ levels is prevalent among ND CKD patients, with differences in prevalence mainly depending on: patient comorbidities; the estimated glomerular filtration rate (eGFR) - in studies in the general population, the incidence of hyperkalemia ranges from 2.9 to 40% in patients with an eGFR <30 mL/min/1.73 m^2^ (6); the number of K measurements (7); and baseline sK levels (2). Patients with CKD may be predisposed to HK for a variety of reasons. Major causes include decreased eGFR levels (8) combined with concomitant treatment with RAASi (9, 10), widely used by clinicians to slow CKD progression and as first choice antihypertensive drugs. An important European survey indicated HK being refractory to medical therapy as the main driver for starting dialysis replacement treatment (1, 11). Several studies have indicated sK values >5.0 mEq/L, that deserve treatment, as a trigger of potentially fatal arrhythmias (12). Furthermore, elevated K+ values often lead the clinicians to discontinue nephroprotective therapy with RAASi, thus favoring the progression of CKD towards dialysis (13). The management and treatment of HK therefore becomes a crucial challenge, not only in dialysis patients, but also and, perhaps, above all in ND CKD patients. A recent Japanese retrospective study found that the prevalence of HK was higher in CKD patients than in the population without CKD. However, HK was found only in 8.3 and 11.6% of patients with CKD stage G4 and G5 respectively, confirming the concept that renal compensation mechanisms and especially therapeutic approaches can give excellent results (14). The presence of HK in the patient with CKD significantly increases the economic impact of CKD itself due to a greater number of hospitalizations and the incidence of HK differs across countries, showing a distinct geographical distribution independent of risk factors including age, sex, medication usage and the presence of diabetes mellitus and hypertension, suggesting possible differences in serum K + monitoring as well as clinical practice across Europe (15). Persistent HK results in higher lifetime costs, besides poorer clinical outcomes, that are evident from the early stages 1-3a of CKD (16). A Swedish study has demonstrated that normal sK, defined as <5.0 mEq/L, confirmed upon two outpatient visits, allows a saving of as much as €16,059 per patient, as compared with hyperkalaemia (17). CKD patients, especially those with associated Diabetes Mellitus (DM), are more susceptible to present with K+ disorders, in particular HK due to kidney disease progression or use of renin-angiotensin-aldosterone blockers (18). In comparison with the general population, patients with DM are more susceptible to HK because of many alterations in the diabetic status, such as hyporeninemic hypoaldosteronism, hyperosmolality, insulin deficiency, and the use of drugs to treat comorbidities (19). For these reasons, the management of HK deserves the attention of the clinician in order to optimize the therapeutic strategies for chronic treatment of HK in the ND CKD patient. This challenge is possible using the new K-binding molecules. The aim of this review is to describe the crucial role of the management of HK in CKD, treating the pathophysiological mechanisms that determine HK, and, furthermore, to describe the prognostic impact on mortality and on the progression of renal damage. Since available treatment options have been increasing, we review the evidence regarding the epidemiology, pathogenesis, etiology and classifications for HK in CKD with a focus on K+ exchange resins.

## 2. Materials and methods

In this narrative review, we have included not only original research papers but also other types of papers (trials, reviews, etc.), on the pathophysiological mechanisms that determine HK. We present the results by starting with the pathophysiology of HK in CKD, followed by a review of the studies that describe the prognosis and prediction of HK, its management and the use of the novel hypokalemic agents.

## 3. Mechanisms of hyperkalemia in CKD

**Figure 1:**
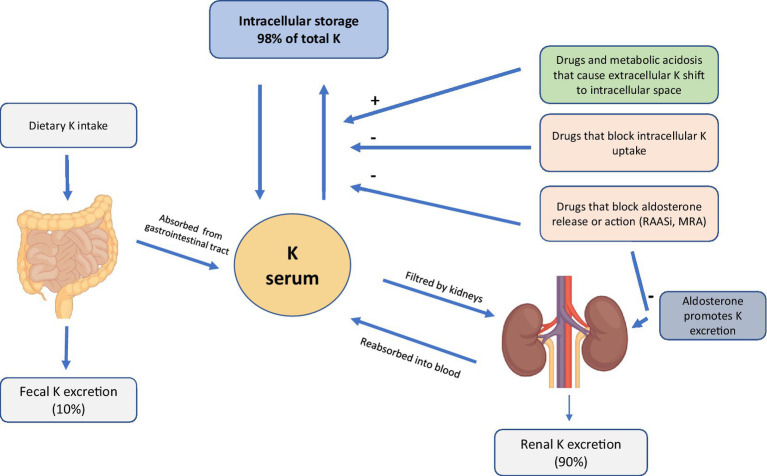
Mechanism of hyperkalemia in CKD.

The pathogenesis of HK is often multifactorial. However, since K+ homeostasis is largely regulated by the kidney (20), renal alterations in ND CKD patients cause increased sK levels due to the failure of K “Adaptation” (21). Thanks to the increased urinary and fecal excretion of K+, sK levels are preserved even in a patient with CKD (22, 23). However, with the progressive deterioration of renal function, generally when the eGFR falls below <20–30 mL/min, this alteration results in a high-risk condition if a patient’s diet is rich in food containing K+ (Figure 1) (22, 23).

Among the causes of HK in ND CKD patients, the widespread use of RAASi may play a crucial role in this category of patients. Currently, the use of RAASi is recommended in patients with albuminuria, because these drugs have a nephroprotective action (24). Moreover, the use of this class of drugs improves cardiovascular prognosis even in patients without proteinuria, but one of the side effects is HK, caused by a reduced urinary excretion of K (24). The incidence of HK in patients receiving RAASi varies between 5 and 40%. The RENAAL study also found that diabetic patients treated with losartan had a higher risk of HK than diabetic patients treated with placebo (25). The reduction in dose and, even worse, the suspension of the drug, can result in giving up an effective weapon in lowering proteinuria levels and slowing the progression of kidney damage (13). In addition, the ND CKD patient often suffers from other comorbidities, such as DM and heart failure with a prevalence of 40%, both of which are frequently associated with HK through different mechanisms (26, 27). DM is identified as an independent predictor of HK. Many mechanisms contribute to a higher risk of developing HK in a DM setting, including impaired K+ excretion, impaired renal tubular function, and a reduced ability to shift K+ into cells. Furthermore, in the patient with CKD and heart failure there is a reduction in the effective arterial blood volume which is followed by reduced glomerular filtration this condition is worsened by any concomitant therapy with RAASi or anti-aldosterone drugs. Interestingly, there is no renal functional reserve in patients with early heart failure but with normal renal function, and both enalapril and losartan can restore a normal vasodilatory response to amino acid infusion in these patients without affecting basal systemic and renal hemodynamics, suggesting a major role of angiotensin II in the development of early renal abnormalities in patients with heart failure (28).

## 4. Hyperkalemia in CKD: a focus on prognosis and prediction

The increase in sKlevels in patients with ND CKD is associated with weakness, paralysis, ventricular arrhythmias, cardiac arrest and an increase in the overall risk of mortality (3). Recently, an Italian study noted that moderate HK is common in ND CKD patients in two visits 12 months apart in a nephrological follow-up. These patients showed a higher risk than the non-HK population of experiencing end stage kidney disease (ESKD), but not higher mortality, over the subsequent 3.6 years (1). It has also been shown in several studies that referral of ND CKD patients to a nephrology center, leads to a more favorable prognosis than for those patients not followed by anephrologist (29, 30). An earlier detection of patients with progressive CKD and interventions to slow progression may have benefits on both kidney and patient survival (31, 32). In patients with stage 5 CKD, the main driver for initiating replacement treatment is HK refractory to medical therapy, as recommended by recent guidelines (33, 34). Furthermore, HK represents a progression factor of CKD up to the ESKD stage (35, 36). Indeed, HK is one of the reasons that most frequently drives the decision to start dialysis treatment in the advanced stages of the disease. One study showed that sK > 6 mEq/L among the ND CKD population was associated with a 30-fold higher mortality risk, while sK > 5 mEq/L was associated with an increase in long-term adverse events preceding ESKD One of the hypotheses is that the finding of elevated sK values prompts the clinician to reduce the dosage or discontinue RAASi therapy (37). An interesting meta-analysis showed that the risk factors associated with HK include co-morbidities, such as congestive heart failure (CHF) and DM and use of drugs, such as diuretics and RAASi (38, 39). The most prominent diuretic is spironolactone, an aldosterone receptor blocker that reduces urinary excretion of K+ by blocking the action of aldosterone, which can then lead to HK. The incidence of HK induced by spironolactone use is significantly higher in patients with CKD very common in diabetic patients due to changes in the microvasculature and when used in combination with ACEi/ARB. Previous studies have found a low prevalence of HK in patients with normal kidney function and markedly elevated frequencies in various cohorts with CKD, especially in patients with DM, in those with more advanced stages of CKD (40). Kovesdy et al. demonstrated that patients with higher levels of albuminuria had a higher prevalence of HK. This finding has important practical relevance, because patients with higher levels of albuminuria often benefit from therapy with RAASi, to slow progression of CKD (24, 41) which also increases the risk of HK (42). The post-hoc analysis of RENAAL found that in patients with DM, the administration of losartan increased the incidence of HK, regardless of renal function.

Furthermore, the risk of HK was found to be increased in heart failure patients and diabetic patients who were receiving combined RAASi or angiotensin receptor blockers (ARB)/angiotensin-converting enzyme inhibitor (ACEi) and anti-aldosterone therapy, and hence serum K+ and renal function should be frequently monitored in such patients (43). The positive effects of losartan’s nephroprotection were partly balanced by the negative effect of the increase in sK values (44). A recent observational study in an Italian cohort examined the cardiorenal prognosis in 2443 patients with CKD, and RAASi were prescribed in 79% of patients. At a median follow-up of 3.6 years, subjects with new onset or persistent HK had an increased risk of entering dialysis by approximately 30%, with a 50% higher risk in patients who were unable to enter dialysis due to HK onset or had suspended RAASi therapy (1). European Society of Cardiology (ESC) Guidelines for the diagnosis and treatment of acute and chronic heart failure recommend concerted efforts to resume RAASi therapy in patients with CHF, even after episodes of severe HK (sK > 6 mEq/L), once HK was treated and precautions taken to monitor sK levels. A 2015 network meta-analysis revived the concept of combined RAAS-blockade as an effective approach to prevent ESKD among patients with diabetic nephropathy (45). Two previous studies had already shown that double blockade with ACEi and ARB increased the risk of episodes in acute kidney injury and HK (46, 47). It appears useful to manage HK by optimizing dietary and drug therapy.

## 5. Management of hyperkalemia in CKD

In ND CKD patients it is essential to treat chronic HK by optimizing dietary and drug therapy.

### 5.1. Nutritional approach

First of all a nutritional approach, in changing the dietary regimen by reducing the intake of foods with a high content of K+ (2–3 gr/day; Figure 1), sodium (Na+; 5–6 gr/day), phosphates (<700 mg/day) and proteins, may be used in order to delay the start of dialysis, while ensuring an adequate caloric intake (30–35 Kcal/kg/day) (48).

A diet rich in fiber (20 gr/day) reduces the acid load and promotes intestinal transit. In fact, although these foods could increase sK levels, the correction of metabolic acidosis and the prevention of constipation help to counteract HK (49). Hence, the hypothesis that constipation is one of the main causes of HK appears valid, and is reinforced by observation that it is rare to find HK in patients with ND CKD on a vegetarian diet (50, 51). International guidelines advise a K+ intake for the ND CKD patient comparable to that of the general population as long as high K+ values are not found (52). However, a recent review recommends an intake of 4.7 gr/day in the early stages of CKD and 2–3 gr/day in the more advanced stages or with an sK > 5.3 mEq/L (53). Studies have shown that even in the advanced stages of CKD, diet remains a crucial step in the prevention and treatment of HK (54). It is advisable to inform the patient about the preferred types of foods, the cooking methods and demineralizing foods, i.e., avoidance of” hidden” K + in some foods and in some additives and low-Na salt substitutes (55). Current data do not confirm that a strict restriction of fruit and vegetable intake translates into a real benefit and control of K+ (56).

### 5.2. Medical therapy

However, in addition to dietary measures, to optimize the control of sK, it may still be necessary to start pharmacological treatment using diuretic therapy, suggested in the types of HK associated with hypervolemia, and sodium bicarbonate, if metabolic acidosis coexists, at a dosage of 2–3 gr/day. Sodium bicarbonate, however, can lead to increased blood pressure and fluid retention in patients with advanced CKD (57). When dietary restrictions are not sufficient and previous therapeutic attempts have failed, K binders are used to maintain an sK < 5.5 mEq/L. These are mainly represented by the cation exchange resins sodium polystyrene sulfonate (SPS), calcium polystyrene sulfonate (CPS), patiromer and sodium zirconium cyclosilicate (SZC) which capture K in the intestinal tract and eliminate it. Among them, the longest available resin on the market, approved by the Food Drug Administration (FDA) in 1958, and most widely used is undoubtedly SPS, a resin that exchanges Na + with K+, calcium (Ca2+) and ammonium and acts on the distal part of the colon. This resin is effective in lowering serum K levels in patients with early stage CKD and mild HK (58), causing watery diarrhea and taking a long time to act. However, in 2009, the same FDA highlighted an increased risk of gastrointestinal adverse events related to the use of this resin as important side effects: poor gastrointestinal tolerability, hypocalcemia, hypomagnesemia and, albeit less frequently, it can cause intestinal necrosis, ischemic colitis and intestinal perforation (59). These data were confirmed by a systematic 2013 review (60) which highlighted transmural necrosis, with increased mortality, among the most frequent histopathological lesions of the colon in patients receiving SPS therapy. This effect is explained by the deposition of SPS crystals in the colon mucosa, which persist even after discontinuation of therapy (61). A recent study has paid particular attention to ND CKD patients with an eGFR <30 mL/min, as they are at greater risk of bleeding from the digestive tract (62). Another limitation of this drug is owing to the Na content which could aggravate hypervolemia, edema and hypertension (63). Another resin used is CPS which acts in the intestine by exchanging K with *Ca.* The most frequent side effects are constipation, abdominal pain, hypercalcemia and hypercalciuria. Furthermore, since this resin can cause distension of the intestinal loops, it is contraindicated in patients with intestinal obstruction. The advantage over SPS is that CPS does not cause Na retention, although SPS has twice the K reabsorption capacity of CPS. However, CPS is less used in the nephrological environment. A study on long-term efficacy in the treatment of mild HK in ND CKD patients has recently been published, demonstrating its efficacy and safety (64). For many years SPS and CPS remained the only K binders available to the clinician. In 2015, two new resins, SZC and patiromer, were approved. These two new resins appear to be of great use in optimally controlling K levels, with less risk of dangerous and more serious adverse events.

### 5.3. Novel hypokalemic agents (patiromer and SZC)

Compared with the old SPS and CPS resins, patiromer and SZC appear to be very interesting because they show lower gastrointestinal toxicity and lower risk of HK. Specifically, patiromer is made up of an anionic polymer and a calcium-sorbitol complex that stabilizes the molecule and allows easier transit in the gastrointestinal tract. The polymer binds K+ mainly in the colon and eliminates it via the faecal route in a dose dependent manner. Recently available in Italy, it was approved by the FDA in 2015 and several clinical trials have been performed to test its efficacy and safety (Table 1).

**Table 1:** Major clinical trials evaluating patiromer for the treatment of hyperkalemia.

Study	Population	Primary end point(s)	Intervention	Major findings
PEARL-HF: Phase II, prospective, randomized, double-blind, placebo-controlled, parallel-group clinical trial	Patients with HF and HK or CKD on aldosterone-antagonist therapy (*n* = 105)	Mean change in K concentration from baseline to the end of the study (day 28)	Patiromer 15 g twice daily versus placebo for 4 weeks	Patiromer lowered serum K+ levels with a difference between groups of −0.45 mEq/L; a lower incidence of HK and a higher proportion of patients on spironolactone 50 mg
AMETHYST-DN: Phase II, prospective, randomized, open-label, dose-ranging clinical trial	Patients with HK and CKD on RAASI therapy (*n* = 306)	Mean change in K concentration from baseline to week 4 or prior to dose titration	Patiromer 4.2 g, 8.4 g, or 12.6 g PO twice daily for mild HK; 8.4 g, 12.6 g PO twice daily for moderate HK versus placebo	Among patients with hyperkalemia and diabetic kidney disease, patiromer starting doses of 4.2 to 16.8 g twice daily resulted in statistically significant decreases in Kl after 4 weeks of treatment, lasting through 52 weeks
OPAL-HK: Phase III prospective clinical trial with a single group, singleblind initial treatment phase and a randomized, singleblind, placebo-controlled withdrawal phase Phase I, prospective, open-label, single-arm clinical trial RAASI (*n* = 25)	Initial 4-week phase: patients with stage 3 or 4 CKD with K > 5.1 mEq/L on RAASI therapy (n = 243) randomized withdrawal 8-week phase: Eligible patients at the end of week 4 were randomly assigned to continue patiromer or switch to placebo patients at the end of the initial phase who had K ≥ 5.5 mEq/L at baseline (*n* = 107) patients with CKD and hyperkalemia (K 5.5–6.4 mEq/L) stabilized on RAASI	Initial phase: mean change in K concentration from baseline to week 4 Randomized phase: between-group difference in the median change in K concentration over the first 4 weeks or to the earliest visit when K was <3.8 or ≥5.5 mEq/L Change in K concentration from baseline over 48 h; time of onset when mean change in K concentration was significant	Initial phase: patiromer 4.2 g PO for mild HK or 8.4 g PO for moderateto-severe HK twice daily for 4 weeks Randomized phase: continued patiromer at same dose received at week 4 or switched to placebo for 8 weeks patiromer 8.4 g PO twice daily with meals for 2 days	Initial phase: reduction in K concentration of −1.01 ± 0.03 mEq/Randomized phase: The median increase in the K level from baseline of that phase was greater with placebo than with patiromer; a recurrence of HK occurred in 60% of the patients in the placebo group as compared with 15% in the patiromer group through week 8 in patients with CKD and HF who were hyperkalaemic on RAASi, patiromer was well tolerated, decreased serum K(+), and, compared with placebo, reduced recurrent hyperkalaemia.

CKD, chronic kidney disease; HF, heart failure; eGFR, estimated glomerular filtration rate; PO, per os (by mouth); RAASi, renin–angiotensin–aldosterone system inhibitor.

In the AMETHYST-DN study (65) and the OPAL-HK study (66), ND CKD patients were examined, including patients with DM and heart failure, with HK and RAASi therapy treated with patiromer and placebo. A significant reduction in sK was observed after 4 weeks, demonstrating the usefulness of this resin to initiate, titrate and maintain adequate therapy with RAASi. It was also observed during the TOURMALINE study that patiromer can be taken by the patient with the same efficacy and safety at any time of the day at least 3 h after taking other drugs for possible drug interactions (67). The second novel agent is SZC, an inorganic polymer based on zirconium silicate whose molecular structure allows it to bind K selectively with respect to other cations such as Ca2+ and Mg2+ (68, 69). In May 2018, this drug was approved by the FDA, after a phase 2 study and two phase 3 studies were carried out. The phase 2 study showed a significant reduction in sK after 48 h in patients treated with SZC compared with the placebo-treated control group (70). The two phase 3 studies included patients with CKD, CHF and treated with RAASi (71, 72), the most frequent adverse effect being the onset of diarrhea, but rarely edema. It is very interesting to note that in these studies there was a correction of metabolic acidosis and a decrease in azotemia in patients under SZC. The DIALIZE study, a double-blind, placebo-controlled, phase 3b multicenter study, evaluated ZS-9 in the management of HK in hemodialysis patients. This study reported that compared with placebo, SZC significantly increased the proportion of patients who maintained pre-dialysis sK levels of 4.0–5.0 mmol/L during at least 3 of 4 HD treatments following the long interdialytic interval and who did not require urgent rescue therapy (73) (Table 2).

**Table 2:** Major clinical trials evaluating SZC for the treatment of hyperkalemia.

Study	Population	Primary end point(s)	Intervention	Major findings
Phase III, prospective, randomized, double-blind, placebo-controlled, two-stage, dose-ranging clinical trial	Initial phase: outpatients with HK,CKD or diabetes (*n* = 754) Maintenance phase: patients with normokalemia at 48 h were randomly assigned to receive either ZS-9 or placebo once daily on days 3 to 14 (*n* = 543)	Initial phase: rate of change in mean potassium concentration compared to placebo over 48 h maintenance phase: exponential rate of change in the mean serum potassium level at 48 h	Initial phase: ZS9 1.25, 2.5 g, 5 g, or 10 g PO three times daily versus placebo maintenance phase: ZS9 dose from initial phase given once daily before or switched to placebo	Initial phase: mean K-concentration reductions of −0.16% for 2.5 g group, −0.21% for 5 g group, −0.30% for 10 g group, and − 0.09% for placebo maintenance phase: patients with HK who received ZS-9, as compared with those who received placebo, had a significant reduction in K at 48 h, with normokalemia maintained during 12 days of maintenance therapy
HARMONIZE: Phase III, prospective, randomized, double-blind, placebo-controlled clinical trial	Open-label phase: outpatients with HK, CKD or diabetes (*n* = 258) randomized phase: outpatients with HK, CKD or diabetes 48 of the initial phase (*n* = 237)	Change in potassium concentration over 48 h mean potassium concentration in each ZS9 group compared to placebo during days 8–29 of the randomized phase	Open-label phase: ZS9 10 g PO three times daily with meals for 2 days Randomized phase: ZS9 5 g, 10 g, or 15 g PO once daily versus placebo for 28 days	Open-label phase: by 48 h, the mean rate of potassium-concentration reduction was −0.3%; the mean absolute reduction was 1.1 mEq/L normokalaemia was maintained with statistically-significant differences observed, irrespective of dose, for Lokelma vs. placebo in terms of mean potassium levels during days 8–29 of the maintenance phase
DIALIZE: Phase III, randomized, double-blind, placebo-controlled study	Adults with ESRD who were managed by three-times weekly hemodialysis and had predialysis hyperkalemia (n.196)	Change in potassium predialysis during the 4-week stable-dose evaluation during at least three of four hemodialysis treatments after the long interdialytic interval	5 g once daily on non HD days, and titrated towards maintaining normokalemia over 4 weeks, in 5 g increments to a maximum of 15 g versus placebo	41.2% Met the primary end point compared with 1.0% of patients receiving placebo; rescue therapy was required by 2.1% of patients taking ZS-9 versus 5.1% taking placebo

CKD, chronic kidney disease; HF, heart failure; eGFR, estimated glomerular filtration rate; PO, per os (by mouth); RAASI, renin–angiotensin–aldosterone system inhibitor; ESRD, end stage renal disease; HD, hemodialysis. Thus, the novel agents are able to bypass the issues associated with the previous resins.

## 6. Future perspective and conclusions

A crucial role in the HK prevention strategy could be a stricter monitoring of sK values in the renal clinic. However, there are currently no guidelines stating exactly how many sK measurements are needed to define clinically meaningful HK. Recent randomized studies have considered at least two measurements of the sK to identify the patients to be enrolled for the initiation of treatment of HK (65). NICE guidelines recommend sK dosing in CKD patients before initiating RAASi therapy, between the first and second weeks of therapy, and after each dose increase. Similar indications are also proposed by the KDIGO regarding the management of CKD (32), considering the higher prevalence of HK in patients with lower eGFR and on RAASi therapy. In these patients, an evaluation of the sK at least every 6 months seems appropriate to promptly identify chronic HK conditions and to increase the dosage of RAASi without exposing the patient to risk. Therefore, it is advisable to measure sK during the first nephrological visit and at subsequent visits. If sK values>5 mmol/L are found in CKD patients, therapeutic dietary and pharmacological strategies should be considered to obtain sK values in an optimal range between 4 and 4.5 mmol/L. The therapeutic options currently available for the treatment of HK are a challenge for the clinician because they are limited to the use of loop diuretics, sodium bicarbonate and K binders such as SPS, which are ineffective and burdened with poor tolerability for side effects and poor palatability. The new resins, patiromer and SZC (73, 74), represent promising weapons in the long-term treatment (both safe) of HK, the main obstacle to the optimization of nephroprotection from RAASi. It has been recently suggested that in patients with acute HK, SZC is the drug of choice due to its more rapid reduction of sK levels; on the other hand, among patients with chronic HK, patiromer appears to be the drug of choice because SZC increases Na absorption leading to an increase in edema (75). Concurrent use of patiromer and high-dosemineral receptor antagonists (MRAs) reduces the risk of recurrent HK (76). Moreover, there is ample evidence that discontinuation/reduction of RAASi therapies is associated with increased adverse outcomes in CKD patients (77, 78).

More clinical trials should test novel drugs, and to see whether the combination of RAASi, novel MRAs, sodium-glucose cotransporter-2 inhibitors (SGLT2-i) and hypokalemic agents will be able to guarantee an improved prognosis. In particular, SGLT2-i use reduced the risk of hyperkalemia because these drugs are known to enhance K+ excretion by the kidney through a combination of mechanisms and are also known to give cardiorenal protection in patients with CKD. Among patients with DM and CKD treated with RAAS inhibitors, recent studies have shown that SGLT2 inhibition with canagliflozin may also reduce the risk of HK without increasing the risk of hypokalemia (79). Moreover, in patients treated with finerenone, a novel nonsteroidal MRA, the onset of HK was twice as high and only 2.3% of patients discontinued the drug due to high sK levels, and no fatal HK was observed (74). In patients with heart failure, RAASi and K-sparing diuretics reduce the risk of death by 15–30%, however, only 25–45% of patients are able to continue treatment at the optimal dosage because of the high risk of developing HK. Further studies will clarify the cardio-renal prognosis of HF and CKD patients with HK during RAASi therapy.

Although, HK has long been considered as a reason for down titration, discontinuation or non-prescription of RAASi, it has not been addressed as a major topic in the literature yet. Further research could explain the impact of RAASi suspension, because of HK, and additional studies could also evaluate its long-term prognostic impact. CKD patients are at higher risk of developing HK because of their comorbidities and concomitant drug therapies, including RAASi. Moreover, further investigations will be required to assess the longer-term prognostic impact of the chronic management of HK. The role of dietary K+ intake in cardiorenal protection needs to be further investigated too; hence, randomized controlled trials of high dietary K+ intake in all the stages of CKD are needed to uncover the relationships and determinants of mortality in this high-risk population. Evidence supporting clinical decision-making for new K+ binders to treat chronic HK in adults with CKD is not strong yet. Indeed, based on existing research, it has not yet been established which resin is the best, so that further studies are needed to evaluate the side effects in long term treatments, particularly for the ones associated with major gastrointestinal symptoms. We cannot be certain about the best antihyperkalemic treatment for patients with CKD. Finally, we underline the need for more information from clinical studies that involve a larger number of patients receiving treatment over long term, so that the combination of these data will provide a useful tool for better clinical and therapeutic decisions.

## Author contributions

DC, GP, RS, and MA: conceptualization, formal analysis, data curation, and writing—review and editing. DC, GP, MP, NI, TF, MZ, FA, GC, DB, GS, UB, LN, PC, RS, and MA: methodology, validation, investigation, writing—original draft preparation, and visualization. MA: supervision. All authors contributed to the article and approved the submitted version.

## Conflict of interest

The authors declare that the research was conducted in the absence of any commercial or financial relationships that could be construed as a potential conflict of interest.

## Publisher’s note

All claims expressed in this article are solely those of the authors and do not necessarily represent those of their affiliated organizations, or those of the publisher, the editors and the reviewers. Any product that may be evaluated in this article, or claim that may be made by its manufacturer, is not guaranteed or endorsed by the publisher.
